# Supplementary material for: Study of a Waste Kaolin as Raw Material for Mullite Ceramics and Mullite Refractories by Reaction Sintering
Source: Materials (Basel). 2022 Jan 13;15(2):583. doi: 10.3390/ma15020583 (PMC8780162; doi:10.3390/ma15020583)
Supplement: Supplementary file 1 [file materials-15-00583-s001.zip › materials-1536115-supplementary.pdf]

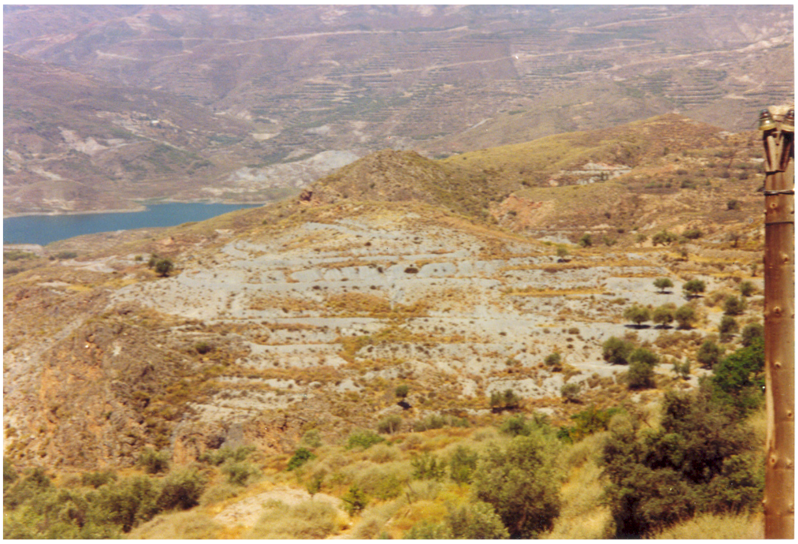

Photography 1: A view of the kaolin deposit.

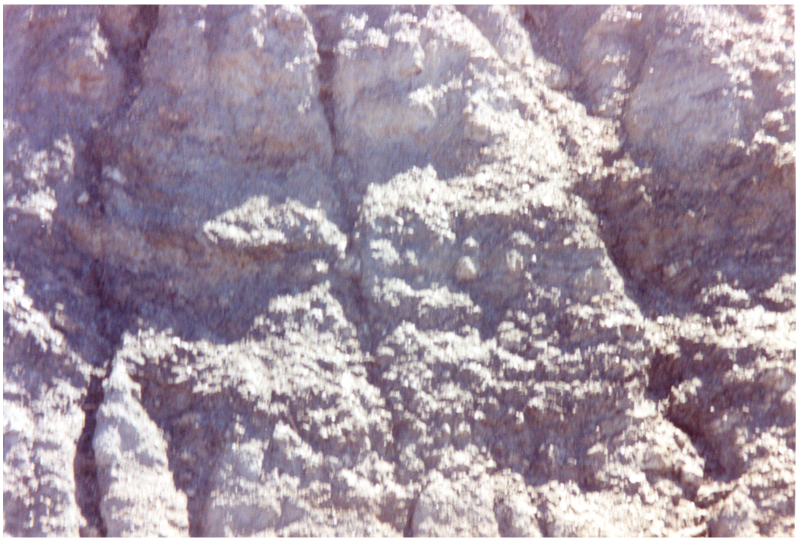

Photography 2: Detail of the raw kaolin where the representative sample was selected to be studied in this investigation.
